# Shared signatures of social stress and aging in peripheral blood mononuclear cell gene expression profiles

**DOI:** 10.1111/acel.12239

**Published:** 2014-06-23

**Authors:** Noah Snyder-Mackler, Mehmet Somel, Jenny Tung

**Affiliations:** 1 Department of Evolutionary Anthropology, Duke UniversityBox 90383, Durham, NC, 27708, USA; 2 Duke University Population Research InstituteBox 90989, Durham, NC, 27708, USA; 3 Department of Biological Sciences, Middle East Technical University06800, Ankara, Turkey; 4 Department of Biology, Duke UniversityBox 90338, Durham, NC 27708, USA

**Keywords:** aging, gene expression, social stress

## Abstract

Chronic social stress is a predictor of both aging-related disease and mortality risk. Hence, chronic stress has been hypothesized to directly exacerbate the process of physiological aging. Here, we evaluated this hypothesis at the level of gene regulation. We compared two data sets of genome-wide gene expression levels in peripheral blood mononuclear cells (PBMCs): one that captured aging effects and another that focused on chronic social stress. Overall, we found that the direction, although not necessarily the magnitude, of significant gene expression changes tends to be shared between the two data sets. This overlap was observable at three levels: (i) individual genes; (ii) general functional categories of genes; and (iii) molecular pathways implicated in aging. However, we also found evidence that heterogeneity in PBMC composition limits the power to detect more extensive similarities, suggesting that our findings reflect an underestimate of the degree to which age and social stress influence gene regulation in parallel. Cell type-specific data on gene regulation will be important to overcome this limitation in the future studies.

The major causes of chronic social stress—low social status, social isolation, and lack of social support—are also linked to higher rates of age-related disease and mortality (House *et al*., 1988; Shaw *et al*., 1999; Sapolsky, 2004; Marmot, 2006; Holt-Lunstad *et al*., 2010). This observation has given rise to the hypothesis that social stress influences the aging process, potentially by affecting the same biological pathways that change during aging. This idea predicts, first, that biomarkers of social stress should also be biomarkers of aging and, second, that the direction of social stress effects on these biomarkers should recapitulate changes with age (Bauer, 2008). Both predictions are supported for a few well-characterized biomarkers, such as IL-6 and telomerase protein levels (Epel *et al*., 2004; Piazza *et al*., 2010; Needham *et al*., 2012; Zalli *et al*., 2014). However, we do not yet know the extent to which these patterns hold more broadly—information that is key for understanding how social stress impacts the aging process.

Here, we compared two previously published data sets to investigate the relationship between chronic social stress and aging for thousands of genes simultaneously. Both data sets measured genome-wide gene expression levels in peripheral blood mononuclear cells (PBMCs). The first, a study of 1240 humans 15–94 years old, captured the effect of age on gene expression (Göring *et al*., 2007; Hong *et al*., 2008). The second involved experimental manipulation of dominance rank (i.e., social status) in 49 rhesus macaques and allowed us to identify genes associated with the response to rank-induced chronic social stress (Tung *et al*., 2012). Importantly, the physiological consequences of both aging and social stress in nonhuman primates often parallel those observed in humans (Roth *et al*., 2004; Sapolsky, 2005), including at the level of gene expression (Somel *et al*., 2010; Tung & Gilad, 2013). Thus, by comparing gene expression levels between the two data sets, we were able to test whether social stress recapitulates the effects of aging.

To do so, we focused on the set of genes (*n* = 4252) included in both data sets. Overall, we found a significant enrichment of genes that were either consistently upregulated or consistently downregulated in both older and lower status individuals (odds ratio = 1.37, Fisher’s exact test, FET, *P* = 3.7 × 10^−7^). This enrichment was even stronger (OR = 2.14, FET *P* = 3.0 × 10^−7^) for the 819 genes that were independently and significantly associated with both variables at a 20% false discovery rate (see Data S1 and Table S2 for similar results using alternative FDR thresholds). Interestingly, some of the genes that were identified in this analysis (Table S1) are also known biomarkers of aging [e.g., *B2M* and *NF-IL6:* (Ershler & Keller, 2000; Annweiler *et al*., 2011)]. In contrast, the magnitude of age and rank effects were not significantly correlated, either across all 4252 genes (Spearman’s *ρ* = 0.065, permutation test *P* = 0.21) or among the 819 genes significantly associated with both age and social stress (Spearman’s *ρ* = 0.062, *P* = 0.36). This observation might be interpreted in two, nonmutually exclusive ways. First, while aging and chronic social stress influence similar genes, their exact impact on these genes may differ. A second likely possibility is that parallels at the level of direction rather than magnitude are more readily detectable across data sets, especially those obtained from different species and using different sampling methods.

In addition to directional similarities at the level of individual genes, social stress and aging could affect similar biological pathways. To test this possibility, we used Gene Ontology (GO) terms (specifically, high-level ‘GO Slim’ categories) to identify functionally related sets of genes that were over-represented among significant genes in each of the two data sets (Ashburner *et al*., 2000). Twenty-nine gene sets were enriched in both cases. Twenty-seven of these ‘co-enriched’ gene categories were similarly affected by social status and age (i.e., either both associated with upregulation or both associated with downregulation with increasing age and lower social status), which was significantly greater than expected by chance (permutation test *P* < 0.0001; Table 1). Only two co-enriched categories, ‘reproduction’ and ‘ATPase activity’, were enriched in both data sets in a manner inconsistent with our motivating hypothesis, no more than expected by chance (*P* = 0.79).

**Table 1:** Co-enriched gene ontology slim categories

Gene ontology category†	OR (FET *P*-value)‡	Rho§ (Spearman’s correlation *P*-value)	Genes compared (*n*)
Upregulated in both			
Signal transduction (BP)	1.20 (*P* = 0.08)		1047
Plasma membrane (CC)	1.22 (*P* = 0.09)		878
Response to stress (BP)	1.19 (*P* = 0.12)		828
Immune system process (BP)	1.13 (*P* = 0.28)		491
Transferase activity, transferring acyl groups (MF)	**1.51 (*****P*** **= 0.29)**	**0.31 (*****P*** **= 0.01)**	**63**
Cytoplasmic membrane-bounded vesicle (CC)	**1.19 (*****P*** **= 0.29)**	**0.15 (*****P*** **= 0.02)**	**251**
Cell morphogenesis (BP)	0.99 (*P* = 0.57)		166
Nucleic acid-binding transcription factor activity (MF)	0.96 (*P* = 0.61)		247
Signal transducer activity (MF)	0.95 (*P* = 0.62)		287
Kinase activity (MF)	0.94 (*P* = 0.64)		267
Homeostatic process (BP)	0.93 (*P* = 0.65)		264
Plasma membrane organization (BP)	0.68 (*P* = 0.82)		16

Downregulated in both			
Nucleus (CC)	**1.46 (*****P*** **= 4.9** × **10**^−**5**^**)**	**0.09 (*****P*** **= 8.4** × **10**^−**5**^**)**	**1794**
Cellular nitrogen compound metabolic process (BP)	**1.53 (*****P*** **= 9.9** × **10**^−**5**^**)**	**0.09 (*****P*** **= 6.3** × **10**^−**4**^**)**	**1308**
Translation factor activity, nucleic acid binding (MF)	**8.19 (*****P*** **= 5.4** × **10**^−**3**^**)**	**0.32 (*****P*** **= 4.7** × **10**^−**2**^**)**	**39**
Nuclear envelope (CC)	**3.02 (*****P*** **= 9.1** × **10**^−**3**^**)**	**0.33 (*****P*** **= 9.6** × **10**^−**4**^**)**	**97**
Nucleolus (CC)	**2.04 (*****P*** **= 1.4** × **10**^−**2**^**)**	**0.16 (*****P*** **= 2.7** × **10**^−**2**^**)**	**186**
RNA binding (MF)	1.81 (*P* = 1.8 × 10^−2^)		270
Translation (BP)	3.01 (*P* = 2.2 × 10^−2^)		111
Protein transporter activity (MF)	2.45 (*P* = 0.16)		38
Ribosome (CC)	2.09 (*P* = 0.17)		63
Nucleoplasm (CC)	1.16 (*P* = 0.22)		510
Methyltransferase activity (MF)	1.81 (*P* = 0.25)		60
Ribonucleoprotein complex assembly (BP)	2.26 (*P* = 0.3)		29
Ribosome biogenesis (BP)	2.57 (*P* = 0.32)		35
DNA binding (MF)	1.08 (*P* = 0.35)		610
mRNA processing (BP)	0.51 (*P* = 0.96)		130

† Co-enriched GO Slim categories for genes that are both upregulated or downregulated with age and low rank. The corresponding GO domain is in parentheses: biological process (BP), cellular component (CC), molecular function (MF). Shaded rows designate categories in which the effects of aging and social stress are more often concordant than not. Categories with significant FET ORs or significant correlations are indicated in bold.

‡ Fisher’s exact test (FET) odds ratio assessing the directional concordance of effects of aging and chronic social stress on gene expression levels for genes within each co-enriched category.

§ Significant correlations (Spearman’s rho) between the effects of aging and chronic social stress on gene expression levels for genes within each co-enriched category.

Because the co-enriched categories were quite broad, we also investigated gene sets linked to well-studied aging-related pathways to test whether they, too, were co-enriched across data sets. Specifically, we investigated gene sets connected to known hallmarks of aging, including inflammation, insulin growth factor signaling, mammalian target of rapamycin (mTOR) signaling, RNA processing, telomere maintenance, mitochondrial senescence, and oxidative stress (López-Otín *et al*., 2013). We found fifteen co-enriched gene sets that were either both upregulated or both downregulated with aging and chronic social stress (Table S3; permutation test *P* = 2.8 × 10^−3^) and none that exhibited the opposite pattern.

We then asked whether genes *within* each category show concordance in the direction of effects across data sets (i.e., concordantly increased or concordantly decreased shifts with older age and lower dominance rank). We identified significant concordance within individual co-enriched GO Slim categories for seven gene sets (Table 1). Furthermore, genes in 22 of the 27 co-enriched GO Slim categories were more often concordant than discordant (binomial test: *P* = 1.5 × 10^−3^). Categories previously linked to aging exhibited a similar pattern (10 of 12, excluding categories with ties; *P* = 3.9 × 10^−2^; Table S3).

Thus, in PBMCs, aging and chronic social stress appear to influence a similar set of both broad categories of genes as well as specific pathways previously implicated in aging. However, we consistently found that directional similarities were more common, and/or easier to detect, than correlations in effect size: only seven of the 27 co-enriched GO Slim categories, and none of the 15 aging-related categories exhibited significant effect size correlations in the predicted direction (Tables 1 and S3). This may be because age and social stress do not alter the same genes within pathways affected by both conditions. Alternatively, discordant changes in PBMC composition between aged and socially stressed individuals might mask parallel changes in gene expression within individual cell types (suggesting that some, but not all, aspects of physiological changes with aging and chronic social stress are shared). Indeed, cell-type composition data from the macaque social stress experiment revealed a significant correlation between cytotoxic T-cell proportions and dominance rank (lower ranking individuals had proportionally fewer of these cells: Tung *et al*., 2012). While T-cell proportions also change with age, they may not do so in a completely parallel manner to that observed with social stress: Depletion of naïve T cells during aging, for example, has been hypothesized to result from accumulated exposure to pathogens over the life course, a mechanism unlikely to be at work in the social stress data set (Larbi *et al*., 2008).

To test whether differences in PBMC composition affected our analysis, we therefore quantified how uniformly each gene was expressed across PBMC cell types. For each gene, we calculated an ‘evenness’ metric, *e*, (Haygood *et al*., 2010) using publicly available gene expression data from each of the five major PBMC cell types in humans (Watkins *et al*., 2009) (SI). We found that, while the subset of genes that were the most evenly expressed (*e* > 0.90; *n* = 555 genes) exhibited strong concordance between the directional effects of aging and social status (OR = 2.45, FET *P* = 1.1 × 10^−6^), this pattern was undetectable among unevenly expressed genes (*e* < 0.90; *n* = 257 genes, OR = 1.27, FET *P* = 0.42). Further, genes in co-enriched categories that were both concordant in direction and significantly correlated between data sets were much more evenly expressed than genes in categories that had concordant, but not significantly correlated effects [Kolmogorov–Smirnov (K-S) test, D = 0.061, *P* = 1.3 × 10^−4^]. In turn, genes in both of these sets were more evenly expressed than genes in categories that had discordant effects (K-S test, D = 0.058, *P* = 0.012; Fig. 1; see Fig. S1 for aging-related categories). Thus, cell-type composition may confound the ability to detect parallels between aging effects and social stress effects for genes with higher levels of tissue-specific expression bias.

**Figure 1:**
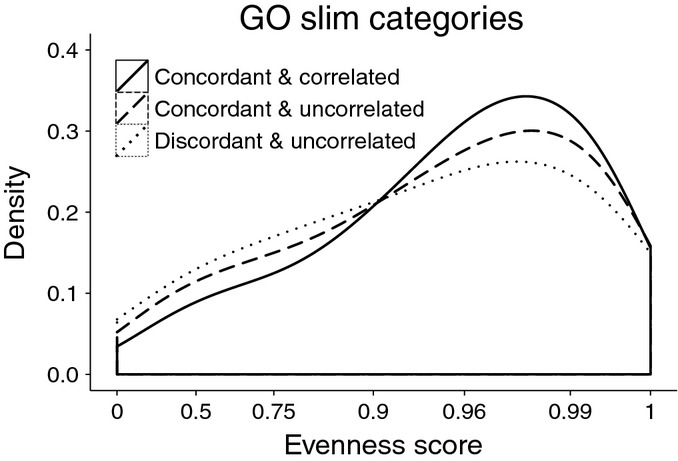
Genes in concordant and significantly correlated co-enriched categories (solid line) are the most evenly expressed across tissues. The ‘evenness score’ measures the degree to which a gene is expressed at the same level across cell types, ranging from 0 (the gene is expressed in only one cell type) to 1 (the gene is equally expressed across the 5 PBMC cell types we considered). Genes in concordant and significantly correlated categories were significantly more evenly expressed than genes in concordant, uncorrelated categories (Kolmogorov–Smirnov (K-S) test, D = 0.061, *P* = 1.3 × 10^−4^), which were in turn significantly more evenly expressed than genes in discordant, uncorrelated categories (K-S test, D = 0.058, *P* = 1.2 × 10^−2^). The *x*-axis is plotted on a negative log scale.

Together, our findings combine to provide support for the hypothesis that social stress broadly recapitulates the physiological effects of aging, at least at the level of gene expression. Thus, this pattern is not restricted to a small set of well-studied biomarkers, but instead appears to be a more general characteristic of cellular physiology. Our analysis also reveals that cell-type-biased gene expression and tissue heterogeneity are likely to hamper the detection of such shared signals, especially at the level of individual genes. Genomic approaches that incorporate controlled, cell-type-specific analyses that focus on aging and chronic social stress effects in the same species—particularly in humans—should help further uncover physiological changes that link social stress to aging and thus social environmental effects to survival and longevity.

## Supporting Information

**Fig. S1** Genes in concordant and significantly correlated co-enriched aging-related categories (solid line) are the most evenly expressed across tissues.

**Table S1** The 472 genes that are significantly associated with older age and lower dominance rank in the same direction.

**Table S2** Comparison of different false discovery rate thresholds.

**Table S3** Co-enriched aging-related gene ontology categories.
