# Supplementary material for: Shared signatures of social stress and aging in peripheral blood mononuclear cell gene expression profiles
Source: Aging Cell. 2014 Jun 23;13(5):954–7. doi: 10.1111/acel.12239 (PMC4172541; doi:10.1111/acel.12239)
Supplement: Supplementary file 2 — Table S1 The 472 genes that are significantly associated with older age and lower dominance rank in the same direction. [file acel0013-0954-sd2.docx]

| Table S1. The 472 genes that are significantly associated with older age and lower dominance rank in the same direction. | | | |
| --- | --- | --- | --- |
|  |  |  |  |
| **Up or down-regulated with increasing age and lower rank** | **Ensembl gene ID** | **Gene Description** |  |
| up-regulated | ENSG00000131759 | retinoic acid receptor, alpha |  |
| up-regulated | ENSG00000197324 | low density lipoprotein receptor-related protein 10 |  |
| up-regulated | ENSG00000141639 | mitogen-activated protein kinase 4 |  |
| up-regulated | ENSG00000160685 | zinc finger and BTB domain containing 7B |  |
| up-regulated | ENSG00000167470 | midnolin |  |
| up-regulated | ENSG00000064393 | homeodomain interacting protein kinase 2; similar to homeodomain interacting protein kinase 2 |  |
| up-regulated | ENSG00000175806 | methionine sulfoxide reductase A |  |
| up-regulated | ENSG00000141002 | transcription factor 25 (basic helix-loop-helix) |  |
| up-regulated | ENSG00000160883 | hexokinase 3 (white cell) |  |
| up-regulated | ENSG00000167987 | vacuolar protein sorting 37 homolog C (*S. cerevisiae*) |  |
| up-regulated | ENSG00000167578 | RAB4B, member RAS oncogene family |  |
| up-regulated | ENSG00000154813 | DPH3, KTI11 homolog *(S. cerevisiae*); DPH3B, KTI11 homolog B (*S. cerevisiae*) |  |
| up-regulated | ENSG00000168071 | coiled-coil domain containing 88B |  |
| up-regulated | ENSG00000075142 | sorcin |  |
| up-regulated | ENSG00000167996 | ferritin, heavy polypeptide 1; ferritin, heavy polypeptide-like 16; similar to ferritin, heavy polypeptide 1; ferritin, heavy polypeptide-like 3 pseudogene |  |
| up-regulated | ENSG00000163464 | interleukin 8 receptor, alpha |  |
| up-regulated | ENSG00000160570 | death effector domain containing 2 |  |
| up-regulated | ENSG00000075239 | acetyl-Coenzyme A acetyltransferase 1 |  |
| up-regulated | ENSG00000100284 | hypothetical LOC100128526; target of myb1 (chicken) |  |
| up-regulated | ENSG00000100345 | myosin, heavy chain 9, non-muscle |  |
| up-regulated | ENSG00000060656 | protein tyrosine phosphatase, receptor type, U |  |
| up-regulated | ENSG00000119048 | ubiquitin-conjugating enzyme E2B (RAD6 homolog) |  |
| up-regulated | ENSG00000197930 | ERO1-like (*S. cerevisiae*) |  |
| up-regulated | ENSG00000180914 | oxytocin receptor |  |
| up-regulated | ENSG00000159388 | BTG family, member 2 |  |
| up-regulated | ENSG00000116514 | ring finger protein 19B |  |
| up-regulated | ENSG00000168256 | NFKB inhibitor interacting Ras-like 2 |  |
| up-regulated | ENSG00000024048 | ubiquitin protein ligase E3 component n-recognin 2 |  |
| up-regulated | ENSG00000217555 | chemokine-like factor |  |
| up-regulated | ENSG00000157551 | potassium inwardly-rectifying channel, subfamily J, member 15 |  |
| up-regulated | ENSG00000183574 | ring finger protein 5; ring finger protein 5 pseudogene 1 |  |
| up-regulated | ENSG00000110876 | selectin P ligand |  |
| up-regulated | ENSG00000134243 | sortilin 1 |  |
| up-regulated | ENSG00000184557 | suppressor of cytokine signaling 3 |  |
| up-regulated | ENSG00000159461 | autocrine motility factor receptor |  |
| up-regulated | ENSG00000127951 | fibrinogen-like 2 |  |
| up-regulated | ENSG00000104312 | receptor-interacting serine-threonine kinase 2 |  |
| up-regulated | ENSG00000144567 | family with sequence similarity 134, member A |  |
| up-regulated | ENSG00000147548 | Wolf-Hirschhorn syndrome candidate 1-like 1 |  |
| up-regulated | ENSG00000153982 | glycerophosphodiester phosphodiesterase domain containing 1 |  |
| up-regulated | ENSG00000116663 | F-box protein 6 |  |
| up-regulated | ENSG00000025708 | thymidine phosphorylase |  |
| up-regulated | ENSG00000126934 | sapiens mitogen-activated protein kinase kinase 2 pseudogene; mitogen-activated protein kinase kinase 2 |  |
| up-regulated | ENSG00000183955 | SET domain containing (lysine methyltransferase) 8 |  |
| up-regulated | ENSG00000111642 | chromodomain helicase DNA binding protein 4 |  |
| up-regulated | ENSG00000160445 | *zer-1* homolog (*C. elegans*) |  |
| up-regulated | ENSG00000165233 | chromosome 9 open reading frame 89 |  |
| up-regulated | ENSG00000004059 | ADP-ribosylation factor 5 |  |
| up-regulated | ENSG00000123689 | G0/G1switch 2 |  |
| up-regulated | ENSG00000003147 | islet cell autoantigen 1, 69kDa |  |
| up-regulated | ENSG00000175040 | carbohydrate (N-acetylglucosamine-6-O) sulfotransferase 2 |  |
| up-regulated | ENSG00000165886 | ubiquitin domain containing 1 |  |
| up-regulated | ENSG00000158710 | transgelin 2 |  |
| up-regulated | ENSG00000108774 | RAB5C, member RAS oncogene family |  |
| up-regulated | ENSG00000067141 | neogenin homolog 1 (chicken) |  |
| up-regulated | ENSG00000163421 | prokineticin 2 |  |
| up-regulated | ENSG00000173334 | tribbles homolog 1 (*Drosophila*) |  |
| up-regulated | ENSG00000205155 | presenilin enhancer 2 homolog (*C. elegans*) |  |
| up-regulated | ENSG00000011132 | amyloid beta (A4) precursor protein-binding, family A, member 3 |  |
| up-regulated | ENSG00000184260 | histone cluster 2, H2ac |  |
| up-regulated | ENSG00000166946 | cyclin D-type binding-protein 1 |  |
| up-regulated | ENSG00000101084 | sapiens chromosome 20 open reading frame 24 |  |
| up-regulated | ENSG00000198055 | G protein-coupled receptor kinase 6 |  |
| up-regulated | ENSG00000206297 | transporter 1, ATP-binding cassette, sub-family B (MDR/TAP) |  |
| up-regulated | ENSG00000125505 | membrane bound O-acyltransferase domain containing 7 |  |
| up-regulated | ENSG00000100307 | chromobox homolog 7 |  |
| up-regulated | ENSG00000163221 | S100 calcium binding protein A12 |  |
| up-regulated | ENSG00000169429 | interleukin 8 |  |
| up-regulated | ENSG00000163220 | S100 calcium binding protein A9 |  |
| up-regulated | ENSG00000171476 | HOP homeobox |  |
| up-regulated | ENSG00000179335 | CDC-like kinase 3 |  |
| up-regulated | ENSG00000104133 | spastic paraplegia 11 (autosomal recessive) |  |
| up-regulated | ENSG00000179526 | SHANK-associated RH domain interactor |  |
| up-regulated | ENSG00000196449 | yrdC domain containing (*E. coli*) |  |
| up-regulated | ENSG00000184922 | formin-like 1 |  |
| up-regulated | ENSG00000172216 | nuclear factor for interleukin 6 |  |
| up-regulated | ENSG00000166710 | beta-2-microglobulin |  |
| up-regulated | ENSG00000174871 | cornichon homolog 2 (*Drosophila*) |  |
| up-regulated | ENSG00000184271 | POU class 6 homeobox 1 |  |
| up-regulated | ENSG00000107796 | actin, alpha 2, smooth muscle, aorta |  |
| up-regulated | ENSG00000163660 | cyclin L1 |  |
| up-regulated | ENSG00000164691 | T-cell activation RhoGTPase activating protein |  |
| up-regulated | ENSG00000137841 | phospholipase C, beta 2 |  |
| up-regulated | ENSG00000137842 | transmembrane protein 62 |  |
| up-regulated | ENSG00000143970 | additional sex combs like 2 (*Drosophila*) |  |
| up-regulated | ENSG00000072694 | Fc fragment of IgG, low affinity IIb, receptor (CD32); Fc fragment of IgG, low affinity IIc, receptor for (CD32) |  |
| up-regulated | ENSG00000113732 | sapiens ATPase, H+ transporting, lysosomal 9kDa, V0 subunit e1 |  |
| up-regulated | ENSG00000130363 | radial spoke 3 homolog (*Chlamydomonas*) |  |
| up-regulated | ENSG00000123143 | protein kinase N1 |  |
| up-regulated | ENSG00000054983 | galactosylceramidase |  |
| up-regulated | ENSG00000166925 | TSC22 domain family, member 4 |  |
| up-regulated | ENSG00000166920 | sapiens chromosome 15 open reading frame 48 |  |
| up-regulated | ENSG00000168487 | bone morphogenetic protein 1 |  |
| up-regulated | ENSG00000137312 | flotillin 1 |  |
| up-regulated | ENSG00000165030 | nuclear factor, interleukin 3 regulated |  |
| up-regulated | ENSG00000166135 | hypoxia inducible factor 1, alpha subunit inhibitor |  |
| up-regulated | ENSG00000173621 | leucine rich repeat and fibronectin type III domain containing 4 |  |
| up-regulated | ENSG00000185022 | v-maf musculoaponeurotic fibrosarcoma oncogene homolog F (avian) |  |
| up-regulated | ENSG00000196923 | PDZ and LIM domain 7 (enigma) |  |
| up-regulated | ENSG00000104973 | mediator complex subunit 25 |  |
| up-regulated | ENSG00000206177 | hemoglobin, mu |  |
| up-regulated | ENSG00000197081 | insulin-like growth factor 2 receptor |  |
| up-regulated | ENSG00000112664 | nudix (nucleoside diphosphate linked moiety X)-type motif 3 |  |
| up-regulated | ENSG00000165914 | tetratricopeptide repeat domain 7B |  |
| up-regulated | ENSG00000105835 | nicotinamide phosphoribosyltransferase |  |
| up-regulated | ENSG00000206172 | hemoglobin, alpha 2; hemoglobin, alpha 1 |  |
| up-regulated | ENSG00000063169 | glioma tumor suppressor candidate region gene 1 |  |
| up-regulated | ENSG00000143207 | ring finger and WD repeat domain 2 |  |
| up-regulated | ENSG00000101460 | sapiens microtubule-associated protein 1 light chain 3 alpha |  |
| up-regulated | ENSG00000062282 | diacylglycerol O-acyltransferase homolog 2 (mouse) |  |
| up-regulated | ENSG00000149925 | aldolase A, fructose-bisphosphate |  |
| up-regulated | ENSG00000114770 | ATP-binding cassette, sub-family C (CFTR/MRP), member 5 |  |
| up-regulated | ENSG00000163702 | interleukin 17 receptor C |  |
| up-regulated | ENSG00000076053 | RNA binding motif protein 7 |  |
| up-regulated | ENSG00000135365 | PHD finger protein 21A |  |
| up-regulated | ENSG00000112511 | PHD finger protein 1 |  |
| up-regulated | ENSG00000115317 | HtrA serine peptidase 2 |  |
| up-regulated | ENSG00000125753 | vasodilator-stimulated phosphoprotein |  |
| up-regulated | ENSG00000214655 | sapiens KIAA0913 |  |
| up-regulated | ENSG00000090020 | solute carrier family 9 (sodium/hydrogen exchanger), member 1 |  |
| up-regulated | ENSG00000204308 | ring finger protein 5; ring finger protein 5 pseudogene 1 |  |
| up-regulated | ENSG00000142347 | myosin IF |  |
| up-regulated | ENSG00000135842 | family with sequence similarity 129, member A |  |
| up-regulated | ENSG00000103426 | coronin 7 |  |
| up-regulated | ENSG00000013364 | major vault protein |  |
| up-regulated | ENSG00000163297 | anthrax toxin receptor 2 |  |
| up-regulated | ENSG00000123700 | potassium inwardly-rectifying channel, subfamily J, member 2 |  |
| up-regulated | ENSG00000115828 | glutaminyl-peptide cyclotransferase |  |
| up-regulated | ENSG00000168394 | transporter 1, ATP-binding cassette, sub-family B (MDR/TAP) |  |
| up-regulated | ENSG00000137076 | talin 1 |  |
| up-regulated | ENSG00000130592 | lymphocyte-specific protein 1 |  |
| up-regulated | ENSG00000105953 | oxoglutarate (alpha-ketoglutarate) dehydrogenase (lipoamide) |  |
| up-regulated | ENSG00000099985 | oncostatin M |  |
| up-regulated | ENSG00000158545 | zinc finger CCCH-type containing 18 |  |
| up-regulated | ENSG00000162645 | guanylate binding protein 2, interferon-inducible |  |
| up-regulated | ENSG00000136811 | outer dense fiber of sperm tails 2 |  |
| up-regulated | ENSG00000119917 | interferon-induced protein with tetratricopeptide repeats 3 |  |
| up-regulated | ENSG00000156427 | fibroblast growth factor 18 |  |
| up-regulated | ENSG00000064547 | lysophosphatidic acid receptor 2 |  |
| up-regulated | ENSG00000125966 | matrix metallopeptidase 24 (membrane-inserted) |  |
| up-regulated | ENSG00000014216 | calpain 1, (mu/I) large subunit |  |
| up-regulated | ENSG00000166501 | protein kinase C, beta |  |
| up-regulated | ENSG00000147883 | cyclin-dependent kinase inhibitor 2B (p15, inhibits CDK4) |  |
| up-regulated | ENSG00000118046 | serine/threonine kinase 11 |  |
| up-regulated | ENSG00000132589 | flotillin 2 |  |
| up-regulated | ENSG00000120738 | early growth response 1 |  |
| up-regulated | ENSG00000097033 | SH3-domain GRB2-like endophilin B1 |  |
| up-regulated | ENSG00000099308 | microtubule associated serine/threonine kinase 3 |  |
| up-regulated | ENSG00000143751 | chromosome 1 open reading frame 55 |  |
| up-regulated | ENSG00000125779 | pantothenate kinase 2 |  |
| up-regulated | ENSG00000135636 | dysferlin, limb girdle muscular dystrophy 2B (autosomal recessive) |  |
| up-regulated | ENSG00000110719 | T-cell, immune regulator 1, ATPase, H+ transporting, lysosomal V0 subunit A3 |  |
| up-regulated | ENSG00000206379 | flotillin 1 |  |
| up-regulated | ENSG00000177981 | ankyrin repeat and SOCS box-containing 8 |  |
| up-regulated | ENSG00000159111 | mitochondrial ribosomal protein L10 |  |
| up-regulated | ENSG00000147894 | chromosome 9 open reading frame 72 |  |
| up-regulated | ENSG00000140105 | tryptophanyl-tRNA synthetase |  |
| up-regulated | ENSG00000129245 | fragile X mental retardation, autosomal homolog 2 |  |
| up-regulated | ENSG00000185883 | ATPase, H+ transporting, lysosomal 16kDa, V0 subunit c |  |
| up-regulated | ENSG00000081181 | arginase, type II |  |
| up-regulated | ENSG00000100106 | TRIO and F-actin binding protein |  |
| up-regulated | ENSG00000107960 | oligonucleotide/oligosaccharide-binding fold containing 1 |  |
| up-regulated | ENSG00000140379 | BCL2-related protein A1 |  |
| up-regulated | ENSG00000123329 | Rho GTPase activating protein 9 |  |
| up-regulated | ENSG00000075151 | eukaryotic translation initiation factor 4 gamma, 3 |  |
| up-regulated | ENSG00000059728 | MAX dimerization protein 1 |  |
| up-regulated | ENSG00000188603 | ceroid-lipofuscinosis, neuronal 3 |  |
| up-regulated | ENSG00000111540 | RAB5B, member RAS oncogene family |  |
| up-regulated | ENSG00000117410 | ATPase, H+ transporting, lysosomal 21kDa, V0 subunit b |  |
| up-regulated | ENSG00000206480 | flotillin 1 |  |
| up-regulated | ENSG00000120129 | dual specificity phosphatase 1 |  |
| up-regulated | ENSG00000149577 | SID1 transmembrane family, member 2 |  |
| up-regulated | ENSG00000169372 | CASP2 and RIPK1 domain containing adaptor with death domain |  |
| up-regulated | ENSG00000129667 | rhomboid 5 homolog 2 (*Drosophila*) |  |
| up-regulated | ENSG00000140983 | ras homolog gene family, member T2 |  |
| up-regulated | ENSG00000148180 | gelsolin (amyloidosis, Finnish type) |  |
| up-regulated | ENSG00000142208 | v-akt murine thymoma viral oncogene homolog 1 |  |
| up-regulated | ENSG00000173262 | solute carrier family 2 (facilitated glucose transporter), member 14 |  |
| up-regulated | ENSG00000025039 | Ras-related GTP binding D |  |
| up-regulated | ENSG00000124731 | triggering receptor expressed on myeloid cells 1 |  |
| up-regulated | ENSG00000197249 | sapiens serpin peptidase inhibitor, clade A (alpha-1 antiproteinase, antitrypsin), member 1 |  |
| up-regulated | ENSG00000156639 | zinc finger, AN1-type domain 3 |  |
| up-regulated | ENSG00000104889 | sapiens ribonuclease H2, subunit A |  |
| up-regulated | ENSG00000166889 | protein associated with topoisomerase II homolog 1 (yeast) |  |
| up-regulated | ENSG00000084676 | nuclear receptor coactivator 1 |  |
| up-regulated | ENSG00000146592 | cAMP responsive element binding protein 5 |  |
| up-regulated | ENSG00000104365 | inhibitor of kappa light polypeptide gene enhancer in B-cells, kinase beta |  |
| up-regulated | ENSG00000105327 | BCL2 binding component 3 |  |
| up-regulated | ENSG00000085514 | paired immunoglobin-like type 2 receptor alpha |  |
| up-regulated | ENSG00000157240 | frizzled homolog 1 (*Drosophila*) |  |
| up-regulated | ENSG00000163162 | ring finger protein 149 |  |
| up-regulated | ENSG00000124201 | zinc finger, NFX1-type containing 1 |  |
| up-regulated | ENSG00000151651 | ADAM metallopeptidase domain 8 |  |
| up-regulated | ENSG00000131196 | nuclear factor of activated T-cells, cytoplasmic, calcineurin-dependent 1 |  |
| up-regulated | ENSG00000132965 | arachidonate 5-lipoxygenase-activating protein |  |
| up-regulated | ENSG00000138772 | annexin A3 |  |
| up-regulated | ENSG00000100614 | protein phosphatase 1A (formerly 2C), magnesium-dependent, alpha isoform |  |
| up-regulated | ENSG00000189190 | zinc finger protein 600 |  |
| up-regulated | ENSG00000122359 | annexin A11 |  |
| up-regulated | ENSG00000121680 | peroxisomal biogenesis factor 16 |  |
| up-regulated | ENSG00000147459 | dedicator of cytokinesis 5 |  |
| up-regulated | ENSG00000049239 | hexose-6-phosphate dehydrogenase (glucose 1-dehydrogenase) |  |
| up-regulated | ENSG00000135926 | transmembrane BAX inhibitor motif containing 1 |  |
| up-regulated | ENSG00000147454 | sapiens solute carrier family 25, member 37 |  |
| up-regulated | ENSG00000213246 | suppressor of Ty 4 homolog 1 (*S. cerevisiae*) |  |
| up-regulated | ENSG00000137642 | sortilin-related receptor, L(DLR class) A repeats-containing |  |
| up-regulated | ENSG00000132024 | coiled-coil and C2 domain containing 1A |  |
| up-regulated | ENSG00000117228 | guanylate binding protein 1, interferon-inducible, 67kDa |  |
| up-regulated | ENSG00000026036 | sapiens tumor necrosis factor receptor superfamily, member 6b, decoy; regulator of telomere elongation helicase 1 |  |
| up-regulated | ENSG00000103196 | sapiens cysteine-rich secretory protein LCCL domain containing 2 |  |
| up-regulated | ENSG00000012779 | arachidonate 5-lipoxygenase |  |
| up-regulated | ENSG00000100393 | E1A binding protein p300 |  |
| up-regulated | ENSG00000118515 | serum/glucocorticoid regulated kinase 1 |  |
| down-regulated | ENSG00000151304 | serum response factor binding protein 1 |  |
| down-regulated | ENSG00000152904 | geranylgeranyl diphosphate synthase 1 |  |
| down-regulated | ENSG00000138381 | asparagine synthetase domain containing 1 |  |
| down-regulated | ENSG00000161057 | proteasome (prosome, macropain) 26S subunit, ATPase, 2 |  |
| down-regulated | ENSG00000163788 | SNF related kinase |  |
| down-regulated | ENSG00000083896 | YTH domain containing 1 |  |
| down-regulated | ENSG00000156482 | ribosomal protein L30 |  |
| down-regulated | ENSG00000152804 | hematopoietically expressed homeobox |  |
| down-regulated | ENSG00000198522 | zinc finger protein 512 |  |
| down-regulated | ENSG00000144029 | mitochondrial ribosomal protein S5 |  |
| down-regulated | ENSG00000089154 | GCN1 general control of amino-acid synthesis 1-like 1 (yeast) |  |
| down-regulated | ENSG00000117519 | calponin 3, acidic |  |
| down-regulated | ENSG00000141646 | SMAD family member 4 |  |
| down-regulated | ENSG00000174953 | DEAH (Asp-Glu-Ala-His) box polypeptide 36 |  |
| down-regulated | ENSG00000163320 | CGG triplet repeat binding protein 1 |  |
| down-regulated | ENSG00000126457 | protein arginine methyltransferase 1 |  |
| down-regulated | ENSG00000139372 | similar to G/T mismatch-specific thymine DNA glycosylase; thymine-DNA glycosylase |  |
| down-regulated | ENSG00000164347 | G elongation factor, mitochondrial 2 |  |
| down-regulated | ENSG00000128708 | histone acetyltransferase 1 |  |
| down-regulated | ENSG00000185009 | adaptor-related protein complex 3, mu 1 subunit |  |
| down-regulated | ENSG00000149474 | CSRP2 binding protein |  |
| down-regulated | ENSG00000156471 | phosphatidylserine synthase 1 |  |
| down-regulated | ENSG00000116649 | spermidine synthase |  |
| down-regulated | ENSG00000114902 | signal peptidase complex subunit 1 homolog (*S. cerevisiae*) |  |
| down-regulated | ENSG00000164941 | integrator complex subunit 8 |  |
| down-regulated | ENSG00000047315 | polymerase (RNA) II (DNA directed) polypeptide B, 140kDa |  |
| down-regulated | ENSG00000106588 | proteasome (prosome, macropain) subunit, alpha type, 2 |  |
| down-regulated | ENSG00000174106 | LEM domain containing 3 |  |
| down-regulated | ENSG00000120053 | glutamic-oxaloacetic transaminase 1, soluble (aspartate aminotransferase 1) |  |
| down-regulated | ENSG00000198719 | delta-like 1 (*Drosophila*) |  |
| down-regulated | ENSG00000115504 | EH domain binding protein 1 |  |
| down-regulated | ENSG00000133561 | GTPase, IMAP family member 6 |  |
| down-regulated | ENSG00000186184 | polymerase (RNA) I polypeptide D, 16kDa |  |
| down-regulated | ENSG00000100883 | sapiens similar to signal recognition particle 54kDa; signal recognition particle 54kDa |  |
| down-regulated | ENSG00000171033 | protein kinase (cAMP-dependent, catalytic) inhibitor alpha |  |
| down-regulated | ENSG00000205765 | chromosome 5 open reading frame 51 |  |
| down-regulated | ENSG00000139343 | small nuclear ribonucleoprotein polypeptide F |  |
| down-regulated | ENSG00000129197 | RPA interacting protein |  |
| down-regulated | ENSG00000104231 | zinc finger, AN1-type domain 1 |  |
| down-regulated | ENSG00000136891 | testis expressed 10 |  |
| down-regulated | ENSG00000138604 | glucuronic acid epimerase |  |
| down-regulated | ENSG00000113263 | IL2-inducible T-cell kinase |  |
| down-regulated | ENSG00000114520 | sorting nexin 4 |  |
| down-regulated | ENSG00000092108 | sec1 family domain containing 1 |  |
| down-regulated | ENSG00000112799 | lymphocyte antigen 86 |  |
| down-regulated | ENSG00000120697 | asparagine-linked glycosylation 5, dolichyl-phosphate beta-glucosyltransferase homolog (S. cerevisiae) |  |
| down-regulated | ENSG00000177764 | zinc finger, CCHC domain containing 3 |  |
| down-regulated | ENSG00000154930 | acyl-CoA synthetase short-chain family member 1 |  |
| down-regulated | ENSG00000036549 | zinc finger, ZZ-type containing 3 |  |
| down-regulated | ENSG00000091483 | fumarate hydratase |  |
| down-regulated | ENSG00000120690 | E74-like factor 1 (ets domain transcription factor) |  |
| down-regulated | ENSG00000078674 | pericentriolar material 1 |  |
| down-regulated | ENSG00000100353 | eukaryotic translation initiation factor 3, subunit D |  |
| down-regulated | ENSG00000140262 | transcription factor 12 |  |
| down-regulated | ENSG00000153046 | chromodomain protein, Y-like |  |
| down-regulated | ENSG00000196683 | translocase of outer mitochondrial membrane 7 homolog (yeast) |  |
| down-regulated | ENSG00000122705 | clathrin, light chain (Lca) |  |
| down-regulated | ENSG00000178896 | exosome component 4 |  |
| down-regulated | ENSG00000062725 | amyloid beta precursor protein (cytoplasmic tail) binding protein 2 |  |
| down-regulated | ENSG00000106049 | 3-hydroxyisobutyrate dehydrogenase |  |
| down-regulated | ENSG00000132781 | mutY homolog (E. coli) |  |
| down-regulated | ENSG00000008083 | jumonji, AT rich interactive domain 2 |  |
| down-regulated | ENSG00000005955 | gametogenetin binding protein 2 |  |
| down-regulated | ENSG00000143033 | metal response element binding transcription factor 2 |  |
| down-regulated | ENSG00000171453 | polymerase (RNA) I polypeptide C, 30kDa |  |
| down-regulated | ENSG00000165819 | methyltransferase like 3 |  |
| down-regulated | ENSG00000055130 | cullin 1 |  |
| down-regulated | ENSG00000134444 | sapiens KIAA1468 |  |
| down-regulated | ENSG00000136270 | transforming growth factor beta regulator 4 |  |
| down-regulated | ENSG00000136271 | DEAD (Asp-Glu-Ala-Asp) box polypeptide 56 |  |
| down-regulated | ENSG00000011405 | phosphoinositide-3-kinase, class 2, alpha polypeptide |  |
| down-regulated | ENSG00000215440 | aminopeptidase-like 1 |  |
| down-regulated | ENSG00000058600 | polymerase (RNA) III (DNA directed) polypeptide E (80kD) |  |
| down-regulated | ENSG00000170242 | ubiquitin specific peptidase 47 |  |
| down-regulated | ENSG00000100823 | APEX nuclease (multifunctional DNA repair enzyme) 1 |  |
| down-regulated | ENSG00000067248 | DEAH (Asp-Glu-Ala-His) box polypeptide 29 |  |
| down-regulated | ENSG00000173085 | coenzyme Q2 homolog, prenyltransferase (yeast) |  |
| down-regulated | ENSG00000173848 | neuroepithelial cell transforming 1 |  |
| down-regulated | ENSG00000117360 | PRP3 pre-mRNA processing factor 3 homolog (*S. cerevisiae*) |  |
| down-regulated | ENSG00000066651 | tRNA methyltransferase 11 homolog (*S. cerevisiae*) |  |
| down-regulated | ENSG00000131508 | ubiquitin-conjugating enzyme E2D 2 (UBC4/5 homolog, yeast) |  |
| down-regulated | ENSG00000104408 | eukaryotic translation initiation factor 3, subunit E |  |
| down-regulated | ENSG00000131652 | THO complex 6 homolog (*Drosophila*) |  |
| down-regulated | ENSG00000116221 | mitochondrial ribosomal protein L37 |  |
| down-regulated | ENSG00000198815 | forkhead box J3 |  |
| down-regulated | ENSG00000185112 | family with sequence similarity 43, member A |  |
| down-regulated | ENSG00000196305 | isoleucyl-tRNA synthetase |  |
| down-regulated | ENSG00000112365 | zinc finger and BTB domain containing 24 |  |
| down-regulated | ENSG00000168564 | sapiens CDKN2A interacting protein |  |
| down-regulated | ENSG00000077232 | DnaJ (Hsp40) homolog, subfamily C, member 10 |  |
| down-regulated | ENSG00000006695 | COX10 homolog, cytochrome c oxidase assembly protein, heme A: farnesyltransferase (yeast) |  |
| down-regulated | ENSG00000096384 | sapiens heat shock protein 90kDa alpha (cytosolic), class B member 1 |  |
| down-regulated | ENSG00000176209 | chromosome 8 open reading frame 40 |  |
| down-regulated | ENSG00000085415 | SEH1-like (S. cerevisiae) |  |
| down-regulated | ENSG00000082898 | exportin 1 (CRM1 homolog, yeast) |  |
| down-regulated | ENSG00000149100 | eukaryotic translation initiation factor 3, subunit M |  |
| down-regulated | ENSG00000102974 | CCCTC-binding factor (zinc finger protein) |  |
| down-regulated | ENSG00000104131 | eukaryotic translation initiation factor 3, subunit J |  |
| down-regulated | ENSG00000116001 | TIA1 cytotoxic granule-associated RNA binding protein |  |
| down-regulated | ENSG00000111252 | SH2B adaptor protein 3 |  |
| down-regulated | ENSG00000100592 | dishevelled associated activator of morphogenesis 1 |  |
| down-regulated | ENSG00000088812 | attractin |  |
| down-regulated | ENSG00000149658 | YTH domain family, member 1 |  |
| down-regulated | ENSG00000077684 | PHD finger protein 17 |  |
| down-regulated | ENSG00000156469 | MTERF domain containing 1 |  |
| down-regulated | ENSG00000105135 | ilvB (bacterial acetolactate synthase)-like |  |
| down-regulated | ENSG00000121022 | COP9 constitutive photomorphogenic homolog subunit 5 (*Arabidopsis*) |  |
| down-regulated | ENSG00000115368 | WD repeat domain 75 |  |
| down-regulated | ENSG00000112200 | zinc finger protein 451 |  |
| down-regulated | ENSG00000147789 | zinc finger protein 7 |  |
| down-regulated | ENSG00000094914 | achalasia, adrenocortical insufficiency, alacrimia (Allgrove, triple-A) |  |
| down-regulated | ENSG00000006194 | zinc finger protein 263 |  |
| down-regulated | ENSG00000143379 | SET domain, bifurcated 1 |  |
| down-regulated | ENSG00000171604 | CXXC finger 5 |  |
| down-regulated | ENSG00000010165 | methyltransferase like 13 |  |
| down-regulated | ENSG00000181610 | mitochondrial ribosomal protein S23 |  |
| down-regulated | ENSG00000029363 | similar to Bcl-2-associated transcription factor 1 (Btf); BCL2-associated transcription factor 1 |  |
| down-regulated | ENSG00000171492 | leucine rich repeat containing 8 family, member D |  |
| down-regulated | ENSG00000137992 | dihydrolipoamide branched chain transacylase E2 |  |
| down-regulated | ENSG00000118482 | PHD finger protein 3 |  |
| down-regulated | ENSG00000105819 | peptidase (mitochondrial processing) beta |  |
| down-regulated | ENSG00000166226 | chaperonin containing TCP1, subunit 2 (beta) |  |
| down-regulated | ENSG00000139505 | myotubularin related protein 6 |  |
| down-regulated | ENSG00000167081 | pre-B-cell leukemia homeobox 3 |  |
| down-regulated | ENSG00000169740 | zinc finger protein 32 |  |
| down-regulated | ENSG00000179833 | SERTA domain containing 2 |  |
| down-regulated | ENSG00000175895 | pleckstrin homology domain containing, family F (with FYVE domain) member 2 |  |
| down-regulated | ENSG00000138032 | protein phosphatase 1B (formerly 2C), magnesium-dependent, beta isoform |  |
| down-regulated | ENSG00000180185 | fumarylacetoacetate hydrolase domain containing 1 |  |
| down-regulated | ENSG00000163002 | nucleoporin 35kDa |  |
| down-regulated | ENSG00000143499 | SET and MYND domain containing 2 |  |
| down-regulated | ENSG00000111581 | nucleoporin 107kDa |  |
| down-regulated | ENSG00000156502 | suppressor of var1, 3-like 1 (*S. cerevisiae*) |  |
| down-regulated | ENSG00000115839 | sapiens RAB3 GTPase activating protein subunit 1 (catalytic) |  |
| down-regulated | ENSG00000114354 | TRK-fused gene |  |
| down-regulated | ENSG00000163510 | CWC22 spliceosome-associated protein homolog (*S. cerevisiae*) |  |
| down-regulated | ENSG00000128228 | stromal cell-derived factor 2-like 1 |  |
| down-regulated | ENSG00000172183 | interferon stimulated exonuclease gene 20kDa |  |
| down-regulated | ENSG00000095564 | BTAF1 RNA polymerase II, B-TFIID transcription factor-associated, 170kDa (Mot1 homolog, *S. cerevisiae*) |  |
| down-regulated | ENSG00000120616 | enhancer of polycomb homolog 1 (*Drosophila*) |  |
| down-regulated | ENSG00000203485 | inverted formin, FH2 and WH2 domain containing |  |
| down-regulated | ENSG00000180376 | coiled-coil domain containing 66 |  |
| down-regulated | ENSG00000058729 | RIO kinase 2 (yeast) |  |
| down-regulated | ENSG00000163607 | GTP-binding protein 8 (putative) |  |
| down-regulated | ENSG00000163811 | WD repeat domain 43 |  |
| down-regulated | ENSG00000112110 | mitochondrial ribosomal protein L18 |  |
| down-regulated | ENSG00000113712 | casein kinase 1, alpha 1 |  |
| down-regulated | ENSG00000181007 | zinc finger protein 82 homolog (mouse) |  |
| down-regulated | ENSG00000101146 | RAE1 RNA export 1 homolog (*S. pombe*) |  |
| down-regulated | ENSG00000100601 | alkB, alkylation repair homolog 1 (*E. coli*) |  |
| down-regulated | ENSG00000138668 | heterogeneous nuclear ribonucleoprotein D (AU-rich element RNA binding protein 1, 37kDa) |  |
| down-regulated | ENSG00000130414 | NADH dehydrogenase (ubiquinone) 1 alpha subcomplex, 10, 42kDa |  |
| down-regulated | ENSG00000124596 | sapiens chromosome 6 open reading frame 130 |  |
| down-regulated | ENSG00000165512 | zinc finger protein 22 (KOX 15) |  |
| down-regulated | ENSG00000156261 | similar to chaperonin containing TCP1, subunit 8 (theta); chaperonin containing TCP1, subunit 8 (theta) |  |
| down-regulated | ENSG00000137692 | DCN1, defective in cullin neddylation 1, domain containing 5 (*S. cerevisiae*) |  |
| down-regulated | ENSG00000107560 | sapiens RAB11 family interacting protein 2 (class I) |  |
| down-regulated | ENSG00000169762 | transmembrane anterior posterior transformation 1 |  |
| down-regulated | ENSG00000140403 | DnaJ (Hsp40) homolog, subfamily A, member 4 |  |
| down-regulated | ENSG00000186918 | zinc finger protein 395 |  |
| down-regulated | ENSG00000135972 | mitochondrial ribosomal protein S9 |  |
| down-regulated | ENSG00000172053 | glutaminyl-tRNA synthetase |  |
| down-regulated | ENSG00000182180 | mitochondrial ribosomal protein S16 |  |
| down-regulated | ENSG00000130811 | eukaryotic translation initiation factor 3, subunit G |  |
| down-regulated | ENSG00000154174 | translocase of outer mitochondrial membrane 70 homolog A (*S. cerevisiae*) |  |
| down-regulated | ENSG00000128791 | twisted gastrulation homolog 1 (*Drosophila*) |  |
| down-regulated | ENSG00000155561 | nucleoporin 205kDa |  |
| down-regulated | ENSG00000118939 | ubiquitin carboxyl-terminal esterase L3 (ubiquitin thiolesterase) |  |
| down-regulated | ENSG00000126249 | programmed cell death 2-like |  |
| down-regulated | ENSG00000213920 | magnesium-dependent phosphatase 1 |  |
| down-regulated | ENSG00000152234 | ATP synthase, H+ transporting, mitochondrial F1 complex, alpha subunit 1, cardiac muscle |  |
| down-regulated | ENSG00000124383 | sapiens M-phase phosphoprotein 10 (U3 small nucleolar ribonucleoprotein) |  |
| down-regulated | ENSG00000198040 | zinc finger protein 84 |  |
| down-regulated | ENSG00000074603 | dipeptidyl-peptidase 8 |  |
| down-regulated | ENSG00000133316 | WD repeat domain 74 |  |
| down-regulated | ENSG00000149806 | Finkel-Biskis-Reilly murine sarcoma virus (FBR-MuSV) ubiquitously expressed |  |
| down-regulated | ENSG00000175575 | proteasomal ATPase-associated factor 1 |  |
| down-regulated | ENSG00000164162 | sapiens anaphase promoting complex subunit 10; anaphase promoting complex subunit 10 pseudogene |  |
| down-regulated | ENSG00000116857 | transmembrane protein 9 |  |
| down-regulated | ENSG00000138081 | F-box protein 11 |  |
| down-regulated | ENSG00000163636 | proteasome (prosome, macropain) 26S subunit, non-ATPase, 6 |  |
| down-regulated | ENSG00000117713 | AT rich interactive domain 1A (SWI-like) |  |
| down-regulated | ENSG00000003393 | amyotrophic lateral sclerosis 2 (juvenile) |  |
| down-regulated | ENSG00000161981 | small nuclear ribonucleoprotein 25kDa (U11/U12) |  |
| down-regulated | ENSG00000155636 | RNA binding motif protein 45 |  |
| down-regulated | ENSG00000032742 | intraflagellar transport 88 homolog (*Chlamydomonas*) |  |
| down-regulated | ENSG00000186106 | ankyrin repeat domain 46 |  |
| down-regulated | ENSG00000142864 | SERPINE1 mRNA binding protein 1 |  |
| down-regulated | ENSG00000196220 | SLIT-ROBO Rho GTPase activating protein 3 |  |
| down-regulated | ENSG00000112237 | cyclin C |  |
| down-regulated | ENSG00000107372 | similar to zinc finger, AN1-type domain 5; zinc finger, AN1-type domain 5 |  |
| down-regulated | ENSG00000141076 | cirrhosis, autosomal recessive 1A (cirhin) |  |
| down-regulated | ENSG00000143106 | proteasome (prosome, macropain) subunit, alpha type, 5 |  |
| down-regulated | ENSG00000089012 | signal-regulatory protein gamma |  |
| down-regulated | ENSG00000122882 | ecdysoneless homolog (*Drosophila*) |  |
| down-regulated | ENSG00000108559 | nucleoporin 88kDa |  |
| down-regulated | ENSG00000163738 | methylenetetrahydrofolate dehydrogenase (NADP+ dependent) 2-like |  |
| down-regulated | ENSG00000129473 | BCL2-like 2 |  |
| down-regulated | ENSG00000101363 | sapiens mannosidase, beta A, lysosomal-like; similar to mannosidase, beta A, lysosomal-like |  |
| down-regulated | ENSG00000013523 | angel homolog 1 (*Drosophila*) |  |
| down-regulated | ENSG00000101365 | isocitrate dehydrogenase 3 (NAD+) beta |  |
| down-regulated | ENSG00000068878 | proteasome (prosome, macropain) activator subunit 4 |  |
| down-regulated | ENSG00000138297 | translocase of inner mitochondrial membrane 23 homolog (yeast); translocase of inner mitochondrial membrane 23 homolog B (yeast) |  |
| down-regulated | ENSG00000137776 | SAFB-like, transcription modulator |  |
| down-regulated | ENSG00000113552 | glucosamine-6-phosphate deaminase 1 |  |
| down-regulated | ENSG00000116044 | nuclear factor (erythroid-derived 2)-like 2 |  |
| down-regulated | ENSG00000146833 | tripartite motif-containing 4 |  |
| down-regulated | ENSG00000137760 | alkB, alkylation repair homolog 8 (*E. coli*) |  |
| down-regulated | ENSG00000055917 | pumilio homolog 2 (*Drosophila*) |  |
| down-regulated | ENSG00000140386 | S-phase cyclin A-associated protein in the ER |  |
| down-regulated | ENSG00000170445 | histidyl-tRNA synthetase |  |
| down-regulated | ENSG00000089022 | sapiens mitogen-activated protein kinase-activated protein kinase 5 |  |
| down-regulated | ENSG00000062194 | GC-rich promoter binding protein 1 |  |
| down-regulated | ENSG00000124459 | zinc finger protein 45 |  |
| down-regulated | ENSG00000131473 | ATP citrate lyase |  |
| down-regulated | ENSG00000171566 | pleiotropic regulator 1 (PRL1 homolog, *Arabidopsis*) |  |
| down-regulated | ENSG00000166557 | transmembrane emp24 protein transport domain containing 3 |  |
| down-regulated | ENSG00000120708 | transforming growth factor, beta-induced, 68kDa |  |
| down-regulated | ENSG00000180530 | nuclear receptor interacting protein 1 |  |
| down-regulated | ENSG00000162222 | tetratricopeptide repeat domain 9C |  |
| down-regulated | ENSG00000023516 | A kinase (PRKA) anchor protein 11 |  |
| down-regulated | ENSG00000112983 | bromodomain containing 8 |  |
| down-regulated | ENSG00000180957 | phosphatidylinositol transfer protein, beta |  |
| down-regulated | ENSG00000162607 | ubiquitin specific peptidase 1 |  |
| down-regulated | ENSG00000130305 | NOL1/NOP2/Sun domain family, member 5 |  |
| down-regulated | ENSG00000111142 | methionyl aminopeptidase 2 |  |
| down-regulated | ENSG00000124207 | CSE1 chromosome segregation 1-like (yeast) |  |
| down-regulated | ENSG00000140391 | tetraspanin 3 |  |
| down-regulated | ENSG00000132341 | RAN, member RAS oncogene family |  |
| down-regulated | ENSG00000134545 | killer cell lectin-like receptor subfamily C, member 1 |  |
| down-regulated | ENSG00000136143 | succinate-CoA ligase, ADP-forming, beta subunit |  |
| down-regulated | ENSG00000185085 | integrator complex subunit 5 |  |
| down-regulated | ENSG00000114956 | deoxyguanosine kinase |  |
| down-regulated | ENSG00000149089 | sapiens APAF1 interacting protein; similar to APAF1 interacting protein |  |
| down-regulated | ENSG00000110435 | pyruvate dehydrogenase complex, component X |  |
| down-regulated | ENSG00000145740 | solute carrier family 30 (zinc transporter), member 5 |  |
| down-regulated | ENSG00000129083 | coatomer protein complex, subunit beta 1 |  |
| down-regulated | ENSG00000166685 | component of oligomeric golgi complex 1 |  |
| down-regulated | ENSG00000127022 | calnexin |  |
| down-regulated | ENSG00000136243 | nucleoporin like 2 |  |
| down-regulated | ENSG00000198492 | YTH domain family, member 2 |  |
| down-regulated | ENSG00000109606 | DEAH (Asp-Glu-Ala-His) box polypeptide 15 |  |
| down-regulated | ENSG00000129480 | sapiens chromosome 14 open reading frame 126 |  |
| down-regulated | ENSG00000204611 | zinc finger protein 616 |  |
| down-regulated | ENSG00000079134 | THO complex 1 |  |
| down-regulated | ENSG00000144895 | eukaryotic translation initiation factor 2A, 65kDa |  |
| down-regulated | ENSG00000181690 | pleiomorphic adenoma gene 1 |  |
| down-regulated | ENSG00000132432 | Sec61 gamma subunit |  |
| down-regulated | ENSG00000117616 | chromosome 1 open reading frame 63 |  |
| down-regulated | ENSG00000115806 | golgi reassembly stacking protein 2, 55kDa |  |
| down-regulated | ENSG00000100528 | cornichon homolog (*Drosophila*) |  |
| down-regulated | ENSG00000126858 | ras homolog gene family, member T1 |  |
| down-regulated | ENSG00000088682 | coenzyme Q9 homolog (*S. cerevisiae*) |  |
| down-regulated | ENSG00000132953 | exportin 4 |  |
| down-regulated | ENSG00000198642 | kelch-like 9 (*Drosophila*) |  |
| down-regulated | ENSG00000143612 | chromosome 1 open reading frame 43 |  |
| down-regulated | ENSG00000154511 | family with sequence similarity 69, member A |  |
| down-regulated | ENSG00000118513 | v-myb myeloblastosis viral oncogene homolog (avian) |  |
| down-regulated | ENSG00000077097 | topoisomerase (DNA) II beta 180kDa |  |
