# Supplementary material for: Shared signatures of social stress and aging in peripheral blood mononuclear cell gene expression profiles
Source: Aging Cell. 2014 Jun 23;13(5):954–7. doi: 10.1111/acel.12239 (PMC4172541; doi:10.1111/acel.12239)
Supplement: Supplementary file 3 — Table S2 Comparison of different false discovery rate thresholds. [file acel0013-0954-sd3.docx]

**Table S2 Comparison of different False Discovery Rate thresholds.**

| **FDR** | **Concordant significant genes^a^** | **Discordant significant genes^b^** | **Odds Ratio** | **rho** | **Co-enriched, concordant GO terms^c^** |
| --- | --- | --- | --- | --- | --- |
| 0.10 | 177 | 140 | 1.85 (p=0.011) | 0.02 (p=0.47) | 12 (p=0.0001) |
| 0.15 | 331 | 241 | 2.19 (p=1.1x10^-5^) | 0.05 (p=0.40) | 17 (p=0.0013) |
| 0.20 | 472 | 347 | 2.14 (p=3.0x10^-7^) | 0.06 (p=0.36) | 27 (p=0.0001) |
| 0.25 | 639 | 469 | 2.03 (p=1.4x10^-8^) | 0.08 (p=0.31) | 50 (p=0.0001) |

^a^ Number of genes that were both significantly upregulated or both significantly downregulated in association with older age and lower social status.

^b^ Number of genes that were significantly upregulated in older individuals and downregulated with low rank (or vice versa).

^c^ Number of GO slim categories that were enriched for genes that were upregulated with age and upregulated with high rank or downregulated with age and downregulated with low rank. p*-*values were calculated using the simulations described in the SI.
