# Supplementary material for: Shared signatures of social stress and aging in peripheral blood mononuclear cell gene expression profiles
Source: Aging Cell. 2014 Jun 23;13(5):954–7. doi: 10.1111/acel.12239 (PMC4172541; doi:10.1111/acel.12239)
Supplement: Supplementary file 4 — Table S3 Co-enriched aging-related gene ontology categories. [file acel0013-0954-sd4.docx]

**Table S3** **Co-enriched Aging-related Gene Ontology categories.**

|  | **Gene Ontology category ^§^** | **FET OR**^¶^ | **rho*** | **Genes compared (n)** |
| --- | --- | --- | --- | --- |
| up-regulated in both | inflammatory response (BP) | 1.79 (p=0.08) | | 138 |
|  | production of molecular mediator involved in inflammatory response (BP) | 5.29 (p=0.38) | | 10 |
|  | insulin receptor signaling pathway (BP) | 1.11 (p=0.54) | | 62 |
|  | cellular response to insulin stimulus (BP) | 0.95 (p=0.63) | | 76 |
|  | acute inflammatory response (BP) | 1.03 (p=0.66) | | 26 |
|  | response to insulin stimulus (BP) | 0.88 (p=0.69) | | 91 |
|  | positive regulation of inflammatory response (BP) | 0.52 (p=0.89) | | 19 |
|  | regulation of insulin receptor signaling pathway (BP) | 0.00 (p=1.0) |  | 10 |
|  | regulation of cellular response to insulin stimulus (BP) | 0.00 (p=1.0) |  | 10 |
|  | chronic inflammatory response (BP) | 0.00 (p=1.0) | -0.86 (p=0.024) | 7 |
|  | negative regulation of insulin receptor signaling pathway (BP) | 0.00 (p=1) |  | 9 |
|  |  |  |  |  |
| down-regulated in both | RNA processing (BP) | 0.95 (p=0.62) | | 217 |
|  | insulin-like growth factor I binding (MF) | 0.94 (p=0.64) | | 199 |
|  | mRNA processing (BP) | 0.58 (p=0.93) | | 132 |
|  | rRNA processing (BP) | 0.00 (p=1.0) |  | 21 |

**^§^** Co-enriched categories for genes that are both up-regulated or down-regulated with age and low rank. The corresponding GO domain is in parentheses: Biological Process (BP) or Molecular Function (MF). Shaded rows designate categories in which the effects of aging and social stress are more concordant than not. Categories with significant FET ORs and correlations indicated in bold.

^¶^ Fisher’s exact test (FET) odds ratio assessing the concordance of effects of aging and chronic social stress on genes within each co-enriched category.

* Significant correlations (Spearman’s rho) between the effects of aging and chronic social stress on genes within each co-enriched category.
