# Supplementary material for: Shared signatures of social stress and aging in peripheral blood mononuclear cell gene expression profiles
Source: Aging Cell. 2014 Jun 23;13(5):954–7. doi: 10.1111/acel.12239 (PMC4172541; doi:10.1111/acel.12239)
Supplement: Supplementary file 5 — Data S1 Supplemental methods. [file acel0013-0954-sd5.docx]

**Supplemental Information for Snyder-Mackler et al, “**Shared signatures of social stress and aging in PBMC gene expression profiles**”**

***Description of the data sets:***

To compare the effects of chronic social stress and aging on gene expression in peripheral blood mononuclear cells (PBMCs), we drew on three previously published gene expression data sets (described below). All three datasets used Illumina gene expression microarrays, which resulted in a high degree of overlap in transcripts measured across studies. To reconcile gene-level gene expression values from the three studies, we linked all probes to Ensembl gene IDs using the Bioconductor package, *biomaRt* (Durinck et al. 2009; Durinck et al. 2005). Where multiple probes were associated with the same Ensembl gene ID we treated the median of the probe-specific expression values for each sample as the gene expression level for the gene in that sample.

To identify genes in the aging and chronic social stress data sets for further analysis, we used a false discovery rate of 20%. This threshold allowed us to maximize our power to detect similarities between aging and chronic social stress, given the noise introduced by using experimental data collected at different times, by different investigators, and on different species (see also other comparisons between aging-related array data sets: e.g., de Magalhães et al. 2009). Importantly, our results on directional concordance, correlation between gene sets, and the excess of co-enriched gene categories are robust to a range of FDR values (from 10% - 25%: Table S2; lower FDR thresholds lead to a severe reduction in the number of analyzable genes).

*Aging data set:*

To measure the effect of age on the transcriptome, we used gene expression data from Göring et al (2007), who measured gene expression levels in 1,240 humans ranging in age from 15-94 years old (mean = 39.3, sd = 16.7; 506 men and 734 women: see Göring et al. 2007 for additional details on the study subjects). These data were generated using the Illumina Sentrix Human Whole Genome (WG-6) Series I BeadChip platform (data available at Array Express accession E-TABM-305). Age is a known predictor of variation in gene expression levels in this data set, as has previously been reported elsewhere (Hong et al. 2008).

To measure the effect of age on gene expression levels, we calculated the correlation (Spearman’s *ρ*) between age and gene expression values for each of the 12,728 genes that we could assign to Ensembl gene IDs. At a false discovery rate (q-value of 20%, corresponding in this data set to a p-value <= 0.21; Storey & Tibshirani 2003), 27% (3500/12728) of gene expression levels were positively correlated with age (i.e., upregulated in older individuals). At the same significance threshold, 27% (3464/12728) of gene expression levels were negatively correlated with age.

*Chronic social stress data set:*

To capture the effect of chronic social stress on the transcriptome, we used gene expression data from Tung et al (2012). This study focused on a non-human primate model of chronic social stress: forty-nine captive female rhesus macaques that varied in social status (i.e., dominance rank: Tung et al. 2012). Specifically, social status was experimentally manipulated in this study via construction of new social groups and sequential introduction of the study subjects into new groups – a process that is highly predictive of subsequent dominance rank (individuals introduced earlier tend to occupy higher ranks: see methods in Tung et al. 2012 and Jarrell et al. 2008 for details on group formation, and Table S1 in Tung et al. 2012 for details on the 49 study subjects). Low ranking individuals were subjected to higher rates of aggression, had reduced control over their environment, and (due to the stability of female macaque social hierarchies) experienced persistent harassment over time. Together, this resulted in a state of chronic stress (Bernstein 1976; Bernstein & Gordon 1974; Bernstein et al. 1974) characterized by decreased GC negative feedback and altered immunological and neurotransmitter regulation (Shively 1998; Jarrell et al. 2008; Tung et al. 2012; Paiardini et al. 2009). Importantly, these hormonal and gene expression effects parallel those associated with chronic social stress in humans (Miller et al. 2007; Tung & Gilad 2013).

Tung et al (2012) used this system to characterize how social status-induced social stress influences gene expression levels, using the Illumina HT-12 v4.0 Expression BeadChip (Gene Expression Omnibus accession GSE33090). Based on this data set, we assigned 5,683 genes to Ensembl gene IDs. We assessed the effect of social status on gene expression using a linear mixed effects model (following the analysis reported in Tung et al. 2012). We corrected for multiple hypothesis testing using the same approach described for the aging data set (Storey & Tibshirani 2003). At a 20% FDR threshold (corresponding to p = 0.11 in this data set), 19% of genes (850/5683) were more highly expressed in low-ranking individuals, while 15% (1062/5683) were more highly expressed in high-ranking individuals.

*Cell type-specific expression data set:*

The HaemAtlas gene expression data set characterized gene expression levels in seven humans for eight purified blood cell types per individual, using Illumina Human WG-6 v2 Expression BeadChips (Watkins et al. 2009: Array Express accession E-TABM-633). In our analysis, we focused on the 5 major cell types found in peripheral blood mononuclear cells: B cells, cytotoxic T cells, helper T cells, Natural Killer (NK) cells, and monocytes, which together make up >98% of PBMCs (Autissier et al. 2010). For each gene and cell type we averaged the gene expression level across the 7 subjects to obtain a measure of each gene’s mean expression level in each of the 5 cell types. For all analyses we used the genes in HaemAtlas that overlapped the genes in both the aging and chronic social stress data sets, resulting in a set of 4,213 genes.

*Intersection of genes in the data sets:*

To test our main hypothesis that the effects of chronic social stress (induced by low social status/dominance rank) recapitulate those of aging, we focused on the 4,252 genes that were measured in both the aging and chronic social stress data sets. Of these genes, 27% (n=1,169) and 20% (n=853) were down-regulated with age and low social status, respectively, and 32% (n=1,359) and 13% (n=565) were up-regulated with age and low social status, respectively.

***Comparison between the effects of age and social stress on gene expression levels***

We first tested if aging and chronic social stress exerted similar directional effects on gene expression across all genes as well as within the subset of genes that were significantly and independently associated with aging and low social status. To do so, we conducted a Fisher’s Exact Test (FET) to test if genes were more likely to be both upregulated or both downregulated by age and low social status than expected by chance. We used a one-tailed test, as we were specifically testing for concordant directions of change rather than discordance (i.e., genes upregulated with age and downregulated with low social status, or vice versa).

Next, we examined whether aging and chronic social stress exerted similar effects on gene expression in both direction and magnitude. To do so, we calculated the overall correlation between the effect sizes in the two studies (Spearman’s *ρ* in the aging data set and *β* for the effect of dominance rank on gene expression from the social stress data set). We assessed the significance of this correlation via permutation. Specifically, we randomly permuted the ages of the subjects in the aging data set, and then re-calculated Spearman’s *ρ* for the permuted age vector and gene expression levels for each gene. This procedure resulted in a new estimated effect size (expected to be near zero) for each gene, which we then correlated with the *β* values obtained from the social stress data set. We calculated the p-value for the correlation between aging effects and social stress effects on gene expression as the proportion of cases (in 10,000 permutations) in which the magnitude of the correlation coefficients relating age effects to social stress effects based on permuted age data was larger than the true correlation.

***Categorical enrichment analysis***

We conducted categorical enrichment analysis using Gene Ontology (GO) terms (Harris et al. 2004) to test if aging and chronic social stress tended to be associated with genes with similar biological functions. The full set of GO terms includes thousands of categories that are arranged hierarchically: “descendant” categories, which contain fewer genes, are embedded within broader, “parent” categories, containing larger sets of genes. To minimize the number of statistical tests we conducted, we chose to focus on “parent” categories. Specifically, we used a set of “GO Slim” categories (http://www.geneontology.org/GO.slims.shtml), which provide an overview of major gene classifications without employing specific descendant terms. We also performed a categorical enrichment analysis on a subset of processes that are associated with the major hallmarks of aging (López-Otín et al. 2013). For this second, more focused analysis we conducted enrichment analysis on the 56 GO categories (in this case, descendant categories, not broad GO Slim parent categories) that contained any of the following search terms: “inflammat*”, “andro*”,”angiote*”,“IL-6”, “insulin-like growth factor”, “TOR”, “telomer*”, “insulin”, “oxidative stress”,”RNA process*”. The results of this analysis are presented in the main text and in table S3 and figure S1. All categorical enrichment analyses were conducted using the hypergeometric test, employing a q-value cutoff of 0.2 for significance.

We first conducted categorical enrichment analysis for the aging data set and the chronic social stress data set separately. In each case, we carried out two analyses: 1) categorical enrichment among genes that were significantly up-regulated with age (or low rank) and 2) categorical enrichment among genes that were significantly down-regulated with age (or low rank). We identified 95 significantly over-represented GO Slim categories in the aging data set and 37 over-represented GO Slim categories in the social status data set. Next, we tested for co-enriched categories, defined as those categories enriched among significant age-associated genes and among significant rank-associated genes. Finally, we used a permutation approach to assess whether the number of co-enriched categories we identified was greater than expected by chance. Specifically, in each of 10,000 iterations, we: 1) permuted age on gene expression levels in the aging data set, 2) re-calculated the correlation between permuted age and gene expression, 3) identified the 3,500 most positively correlated genes and the 3,464 most negatively correlated genes (these numbers corresponded to the observed number of genes significantly positively and negatively correlated with age), 4) conducted a categorical enrichment analysis on each of these two sets of genes, and 5) counted the number of the enriched GO terms from the permuted data that overlapped with the set of enriched GO terms in the social stress data set.

Each permuted data set therefore yielded four numbers, corresponding to the number of co-enriched categories in genes *i)* up-regulated with age and up-regulated with lower rank; *ii)* down-regulated with age and up-regulated with lower rank; *iii)* up-regulated with age and down-regulated with lower rank; and *iv)* down-regulated with age and down-regulated with lower rank. These numbers represented the co-enrichment of GO categories that would be expected in each of these cases by chance. We used the distributions of these values across 10,000 permutations to calculate a p-value estimating the probability that the number of co-enriched categories we observed in the true data was observable by chance. Specifically, we calculated *p* as the proportion of permuted data sets that yielded a larger number of co-enriched categories than in the true data set, distinguishing between cases in which the co-enrichment matched our predictions (cases i and iv above) and cases that did not. We repeated these analyses for the 56 GO categories that were *a priori* chosen for prior associations with aging (table S3).

***Testing for effects of PBMC cell type heterogeneity***

To test whether the lack of gene-level concordances and correlations between aging and social stress effects within co-enriched categories resulted from potential tissue composition effects, we drew upon PBMC-specific expression data from the HaemAtlas (Watkins et al. 2009). We predicted that genes in discordant and uncorrelated co-enriched categories would exhibit more cell type biased gene expression patterns than genes in the concordant, uncorrelated categories, which in turn would be more cell type biased than the concordant and significantly correlated co-enriched categories. We tested how evenly each of these genes was expressed across the five major PBMC subtypes (helper T cells, cytotoxic T cells, B cells, monocytes, and NK cells).

Gene expression evenness (*e*) was calculated following the method of Haygood *et al* (Haygood et al. 2010) using the cell type-specific gene expression data from the HaemAtlas (Watkins et al. 2009). In its simplest form, comparing the expression of a gene in two tissues/cell types, this metric is equal to 1 – *d*, where *d* equals the absolute value of the difference between the gene expression levels in the two tissues, divided by the sum of that gene’s expression in both tissues. This metric is then scaled between 0 and 1, where higher values represent genes that are more equally expressed in both tissues. This metric can be expanded to calculate gene expression evenness across many tissues using the angle of elevation (*ε*), which corresponds to the deviation from a perfectly evenly expressed gene across all of the tissues, and *e* = cos^2^ε. Parallel to the case in two tissues, *e* can then be scaled to a value between 0 and 1, such that a gene expressed in only one cell type receives a value *e*=0, and a gene that is expressed at the same level in all cell types receives a value of *e*=1. After calculating the values of *e* for all genes in the co-enriched categories, we conducted two pairwise comparisons of the distributions of the evenness scores of genes using one-tailed Kolmogorov-Smirnov tests: 1) concordant and correlated genes versus concordant and uncorrelated genes and 2) concordant and uncorrelated genes versus discordant and uncorrelated genes. We then repeated this analysis with the co-enriched aging-related categories. However, because there were no correlated genes in the aging-related categories, we conducted one pairwise comparison between genes in concordant co-enriched categories versus genes in discordant co-enriched categories using a one-tailed Kolmogorov-Smirnov test (Fig. S1).

Autissier P, Soulas C, Burdo TH & Williams KC (2010) Evaluation of a 12-color flow cytometry panel to study lymphocyte, monocyte, and dendritic cell subsets in humans. *Cytometry. A* 77, 410–9.

Bernstein IS (1976) Dominance, aggression and reproduction in primate societies. *J. Theor. Biol.* 60, 459–472.

Bernstein IS & Gordon TP (1974) The function of aggression in primate societies. *Am. Sci.* 62, 304–311.

Bernstein IS, Gordon TP & Rose RM (1974) Aggression and Social Controls in Rhesus Monkey (Macaca mulatta) Groups Revealed in Group Formation Studies. *Folia Primatol.* 21, 81–107.

Durinck S, Moreau Y, Kasprzyk A, Davis S, De Moor B, Brazma A & Huber W (2005) BioMart and Bioconductor: a powerful link between biological databases and microarray data analysis. *Bioinformatics* 21, 3439–3440.

Durinck S, Spellman PT, Birney E & Huber W (2009) Mapping identifiers for the integration of genomic datasets with the R/Bioconductor package biomaRt. *Nat. Protoc.* 4, 1184–1191.

Göring HHH, Curran JE, Johnson MP, Dyer TD, Charlesworth J, Cole SA, Jowett JBM, Abraham LJ, Rainwater DL, Comuzzie AG, Mahaney MC, Almasy L, MacCluer JW, Kissebah AH, Collier GR, Moses EK & Blangero J (2007) Discovery of expression QTLs using large-scale transcriptional profiling in human lymphocytes. *Nat. Genet.* 39, 1208–1216.

Harris MA, Clark J, Ireland A, Lomax J, Ashburner M, Foulger R, Eilbeck K, Lewis S, Marshall B, Mungall C, Richter J, Rubin GM, Blake JA, Bult C, Dolan M, Drabkin H, Eppig JT, Hill DP, Ni L, Ringwald M, Balakrishnan R, Cherry JM, Christie KR, Costanzo MC, Dwight SS, Engel S, Fisk DG, Hirschman JE, Hong EL, Nash RS, Sethuraman A, Theesfeld CL, Botstein D, Dolinski K, Feierbach B, Berardini T, Mundodi S, Rhee SY, Apweiler R, Barrell D, Camon E, Dimmer E, Lee V, Chisholm R, Gaudet P, Kibbe W, Kishore R, Schwarz EM, Sternberg P, Gwinn M, Hannick L, Wortman J, Berriman M, Wood V, de la Cruz N, Tonellato P, Jaiswal P, Seigfried T & White R (2004) The Gene Ontology (GO) database and informatics resource. *Nucleic Acids Res.* 32, D258–61.

Haygood R, Babbitt CC, Fedrigo O & Wray GA (2010) Contrasts between adaptive coding and noncoding changes during human evolution. *Proc. Natl. Acad. Sci.* 107, 7853–7857.

Hong M-G, Myers AJ, Magnusson PKE & Prince J a (2008) Transcriptome-wide assessment of human brain and lymphocyte senescence. *PLoS One* 3, e3024.

Jarrell H, Hoffman JB, Kaplan JR, Berga S, Kinkead B & Wilson ME (2008) Polymorphisms in the serotonin reuptake transporter gene modify the consequences of social status on metabolic health in female rhesus monkeys. *Physiol. Behav.* 93, 807–819.

López-Otín C, Blasco MA, Partridge L, Serrano M & Kroemer G (2013) The hallmarks of aging. *Cell* 153, 1194–217.

De Magalhães JP, Curado J & Church GM (2009) Meta-analysis of age-related gene expression profiles identifies common signatures of aging. *Bioinformatics* 25, 875–81.

Miller GE, Chen E & Zhou ES (2007) If it goes up, must it come down? Chronic stress and the hypothalamic-pituitary-adrenocortical axis in humans. *Psychol. Bull.* 133, 25–45.

Paiardini M, Hoffman J, Cervasi B, Ortiz AM, Stroud F, Silvestri G & Wilson ME (2009) T-Cell Phenotypic and Functional Changes Associated with Social Subordination and Gene Polymorphisms in the Serotonin Reuptake Transporter in Female Rhesus Monkeys. *Brain. Behav. Immun.* 23, 286–293.

Shively CA (1998) Social subordination stress, behavior, and central monoaminergic function in female cynomolgus monkeys. *Biol. Psychiatry* 44, 882–891.

Storey JD & Tibshirani R (2003) Statistical significance for genomewide studies. *Proc. Natl. Acad. Sci.* 100, 9440–9445.

Tung J, Barreiro LB, Johnson ZP, Hansen KD, Michopoulous V, Toufexis D, Michelini KM, Wilson ME & Gilad Y (2012) Social environment is associated with gene regulatory variation in the rhesus macaque immune system. *Proc. Natl. Acad. Sci.* 109, 6490–5.

Tung J & Gilad Y (2013) Social environmental effects on gene regulation. *Cell. Mol. Life Sci.*

Watkins N a, Gusnanto A, de Bono B, De S, Miranda-Saavedra D, Hardie DL, Angenent WGJ, Attwood AP, Ellis PD, Erber W, Foad NS, Garner SF, Isacke CM, Jolley J, Koch K, Macaulay IC, Morley SL, Rendon A, Rice KM, Taylor N, Thijssen-Timmer DC, Tijssen MR, van der Schoot CE, Wernisch L, Winzer T, Dudbridge F, Buckley CD, Langford CF, Teichmann S, Göttgens B & Ouwehand WH (2009) A HaemAtlas: characterizing gene expression in differentiated human blood cells. *Blood* 113, e1–9.

**Fig. S1 Genes in concordant and significantly correlated co-enriched aging-related categories (solid line) are the most evenly expressed across tissues.** The ‘evenness score’ measures the degree to which a gene is expressed at the same level across cell types, ranging from 0 (the gene is expressed in only one cell type) to 1 (the gene is equally expressed across the 5 PBMC cell types we considered). Genes in concordant categories were significantly more evenly expressed than genes in discordant categories (K-S test, D = 0.55, p=2.1 x10^-6^). The x-axis is plotted on a negative log scale.
